# Supplementary material for: Evaluating Germplasm of Cultivated Oat Species from the VIR Collection under the Russian Northwest Conditions
Source: Plants (Basel). 2022 Nov 28;11(23):3280. doi: 10.3390/plants11233280 (PMC9740924; doi:10.3390/plants11233280)
Supplement: Supplementary file 1 [file plants-11-03280-s001.zip › plants-1757944-supplementary.pdf]

plants

Supplementary Materials

Evaluating Germplasm of Cultivated Oat Species from the VIR Collection under the Russian Northwest Conditions

Table 1. List of accessions of *Avena* L. species from the VIR collection used as research material.

VIR Catalog №	Variety	Origin	VIR Catalog №	Variety	Origin
	***Avena sativa* L.**			***A. byzantina* C.K.**	
k-14787	Privet	Russia	k-15368	St-Mateus	Portugal
k-15381	Chakal	Belarus	k-15386	Saron	Ukraine
k-15441	Yantar'	Russia	k-15397	Image	Great Britain
k-15442	Zalp	Russia	k-15435	Mestnyy	Ethiopia
k-15443	Avatar	Russia	k-15445	194h06	Russia
k-15444	Sapsan	Russia	k-15450	55-12	Russia
k-15446	43-12	Russia	k-15464	Kulager	Kazakhstan
k-15447	44-12	Russia	k-15465	Zhorga	Kazakhstan
k-15448	47-12	Russia	k-15470	Rocy	Germany
k-15449	53-12	Russia	k-15471	Steinar	Finland
k-15451	Foma	Russia	k-15472	Symphony	Germany
k-15452	Orfey	Russia	k-15474	Terruf	USA
k-15453	Novosibirskiy 5	Russia	k-15475	C.I. 3326	USA
k-15454	Novosibirskiy 7	Russia	k-15481	URS Corona	Brazil
k-15455	Mutica 1120	Russia	k-15482	URS Guara	Brazil
k-15456	3449-00	Russia	k-15484	URS Guana	Brazil
k-15457	2204-03	Russia	k-15485	URS Tarimba	Brazil
k-15458	3926-05	Russia	k-15486	URS Charrua	Brazil
k-15459	1628-05	Russia	k-15488	URS Torena	Brazil
k-15460	Urman	Russia	k-15489	URS Torena	Brazil
k-15473	Ozon	Germany			
k-15476	CI3300	USA			
k-15483	URS Penca	Brazil			
	***A. abyssinica* Hochst.**			***A. strigosa* Schreb.**	
k-15436	Local	Ethiopia	k-15478	Agro Zebu	Brazil
k-15437	Local	Ethiopia	k-15479	Agro Planalto	Brazil
k-15438	Local	Ethiopia	k-15480	Agro Coxilha	Brazil

Table 2. Weather conditions at Pushkin and Pavlovsk Laboratories of VIR in 2014–2015.

Year	Factor	June		July		August	
		Average	SD	Average	SD	Average	SD
2014	Temperature, t°C	16.6	3.1	22.3	2.5	19.7	4.9
	Rainfall, mm	29.0	3.4	7.1	3.9	23.0	21.7
2015	Temperature, t°C	18.0	1.2	18.4	1.1	19.9	0.8
	Rainfall, mm	8.1	11.0	38.7	13.2	11.8	2.6

Table 3. Protein, oil, starch and *β*-glucan content (% DW) in the studied *Avena* L. accessions from the VIR collection during the two-year study, mean values ± SD.

VIR Catalog №	Protein		Oil		Starch		*β*-Glucans		VIR Catalog №	Protein		Oil		Starch		*β*-Glucans	
	Mean	SD	Mean	SD	Mean	SD	Mean	SD		Mean	SD	Mean	SD	Mean	SD	Mean	SD
	Avena sativa L.									*A. byzantina* C.K.							
k-14787	12.15	0.49	4.95	0.05	38.39	0.60	2.95	0.11	k-15368	12.22	1.42	5.54*	0.33	39.87	3.62	4.26*	0.65
k-15381	12.84	2.23	3.62	0.14	40.58*	5.14	3.59	0.37	k-15386	14.03	0.21	4.24	0.38	32.56	0.15	-	-
k-15441	13.73	1.17	3.71	0.28	35.97	3.40	-	-	k-15397	13.31	1.69	5.66*	0.51	38.67	3.94	-	-
k-15442	12.36	0.93	4.92	0.15	38.36	1.53	-	-	k-15435	12.43	0.85	5.24*	0.24	33.70	2.66	-	-
k-15443	12.79	0.28	4.35	0.17	34.88	1.02	-	-	k-15445	13.23	0.28	4.50	0.22	37.29	1.09	-	-
k-15444	12.43	0.33	4.91	0.12	38.34	0.67	-	-	k-15450	12.10	0.64	3.16	0.09	39.23	4.37	3.50	0.22
k-15446	13.08	0.15	4.98	0.25	36.31	2.07	-	-	k-15464	13.09	1.59	4.22	0.11	38.62	4.49	-	-
k-15447	12.43	0.00	3.48	0.20	33.04	6.62	4.42*	1.57	k-15465	11.93	1.26	3.34	0.26	39.03	5.42	-	-
k-15448	11.21	0.43	4.81	0.13	39.09	3.41	3.40	0.35	k-15470	11.57	0.33	5.18*	0.08	37.71	1.48	3.52	0.26
k-15449	11.57	0.33	4.15	0.10	38.97	4.54	3.52	0.21	k-15471	12.00	1.17	3.97	0.26	39.37	5.92	3.46	0.44
k-15451	12.22	0.93	3.39	0.19	41.66*	4.23	4.54*	0.31	k-15472	11.67	0.96	3.61	0.13	40.88*	5.00	-	-
k-15452	13.01	1.67	5.06	0.34	38.52	2.77	3.74*	0.14	k-15474	17.05*	1.85	4.82	0.26	34.07	6.80	2.93	0.65
k-15453	11.63	0.28	5.48*	0.45	39.05	3.41	3.67	0.45	k-15475	13.80	0.93	5.63*	0.12	40.88*	1.38	3.17	0.45
k-15454	12.80	0.76	3.84	0.35	41.11*	3.24	4.08*	0.32	k-15481	13.95	2.09	3.86	0.15	35.84	3.74	3.52	0.36
k-15455	13.15	0.17	5.21*	0.53	39.50	4.13	-	-	k-15482	14.17	2.00	4.67	0.10	37.92	5.20	3.23	0.13
k-15456	12.84	0.85	4.88	0.10	40.91*	3.07	-	-	k-15484	15.25*	2.93	4.75	0.09	35.05	4.34	3.19	0.05
k-15457	13.15	0.17	4.69	0.20	36.72	6.12	-	-	k-15485	13.88	2.00	4.88	0.64	38.09	1.10	3.42	0.18
k-15458	13.46	0.86	4.55	0.16	37.97	3.90	-	-	k-15486	14.37	2.77	4.36	0.21	36.73	3.81	3.37	0.05
k-15459	12.29	0.83	4.64	0.11	34.57	5.41	-	-	k-15488	15.25*	2.10	4.41	0.27	36.78	0.81	3.29	0.35
k-15460	13.15	1.21	4.31	0.22	37.75	2.41	-	-	k-15489	13.60	0.00	5.52*	0.57	33.74	0.76	-	-
k-15473	12.00	1.50	3.39	0.17	37.40	5.52	3.67	0.31									
k-15476	15.13*	1.08	4.87	0.09	39.10	3.33	2.87	0.15									
k-15483	17.77*	3.02	5.86*	0.19	36.21	3.52	2.69	0.10									
	A. abyssinica Hochst.									*A. strigosa* Schreb.							
k-15436	15.90*	0.86	4.81	0.40	38.48	1.38	2.46**	0.21	k-15478	19.37*	1.52	3.93	0.16	38.29	4.34	2.71	0.29
k-15437	14.78	0.29	4.81	0.43	33.31	1.02	-	-	k-15479	18.80*	0.66	4.23	0.12	35.38	4.30	3.73*	0.56
k-15438	14.89	0.20	5.39*	0.30	33.61	0.31	-	-	k-15480	17.78*	0.83	4.11	0.10	38.11	4.97	2.49**	0.35

* sources of economically valuable traits (high in protein: above 15.0%, oils: above 5.1%, starch: above 40.5%, *β*-glucans: above 3.7%). ** for use in feed production (with *β*-glucans content: below 2.5%).

Table 4. Plant height, panicle length, number of spikelets, number of grains, 1000 grain weight, and seed productivity (grain yield) in the studied *Avena* L. accessions from the VIR collection, mean values ± SD.

VIR Catalog No	PH, cm		LP, cm		NSP, pcs		NG, pcs		W1000, g		SP, g/m²	
	Mean	SD	Mean	SD	Mean	SD	Mean	SD	Mean	SD	Mean	SD
***Avena sativa* L.**												
k-14787	112.5	17.7	17.5	0.7	33.0	2.8	57.5	4.9	38.1*	1.4	375.0*	162.6
k-15381	107.5	10.6	19.3	1.8	44.0	5.7	77.0	2.8	40.5*	1.9	352.5	81.3
k-15441	120.0	0.0	19.0	1.4	35.5	6.4	51.0	15.6	40.2*	4.1	195.0	162.6
k-15442	112.5	24.7	17.5	1.4	26.5	3.5	47.5	10.6	41.3*	1.3	365.0	35.4
k-15443	107.5	10.6	17.8	0.4	32.0	5.7	59.5	7.8	37.7	2.8	392.5*	95.5
k-15444	107.5	24.7	17.5	0.7	30.5	3.5	58.5	4.9	42.7*	1.6	447.5*	46.0
k-15446	130.0	21.2	21.3	0.4	42.5	7.8	68.5	10.6	39.9*	1.3	267.5	17.7
k-15447	120.0	7.1	20.0	0.0	35.5	4.9	52.5	10.6	44.5*	2.3	357.5	123.7
k-15448	122.5	24.7	19.8	1.1	32.5	0.7	59.5	4.9	42.2*	2.7	295.0	91.9
k-15449	130.0	21.2	21.0	2.8	33.0	7.1	53.5	12.0	41.1*	1.5	237.5	180.3
k-15451	107.5	10.6	17.8	0.4	35.0	2.8	54.0	8.5	38.8*	2.5	327.5	279.3
k-15452	115.0	21.2	20.5	0.7	32.5	2.1	48.0	4.2	44.5*	0.0	225.0	21.2
k-15453	110.0	14.1	19.5	2.1	33.0	2.8	54.0	4.2	42.8*	1.0	432.5*	31.8
k-15454	115.0	21.2	19.5	2.1	34.0	4.2	48.5	3.5	44.5*	2.0	200.0	7.1
k-15455	130.0	7.1	23.0	3.5	39.5	12.0	52.0	5.7	39.3*	2.5	190.0	7.1
k-15456	115.0	21.2	18.5	3.5	32.5	6.4	48.0	2.8	44.0*	2.7	285.0	113.1
k-15457	127.5	10.6	19.5	0.7	41.5	7.8	61.0	19.8	40.8*	0.4	402.5*	152.0
k-15458	115.0	21.2	18.0	1.4	36.5	2.1	57.0	2.8	40.6*	2.1	302.5	95.5
k-15459	112.5	17.7	18.8	1.8	34.0	1.4	55.0	0.0	43.3*	1.9	312.5	38.9
k-15460	115.0	21.2	19.8	0.4	26.5	7.8	55.5	12.0	48.0*	2.5	315.0	14.1
k-15473	105.0	14.1	16.8	2.5	34.0	5.7	59.5	4.9	42.4*	0.8	402.5*	3.5
k-15476	122.5	17.7	18.8	0.4	24.0	1.4	39.0	2.8	40.7*	3.3	217.5	88.4
k-15483	97.5	3.5	16.5	0.7	27.5	4.9	41.5	14.8	41.2*	0.8	305.0	233.3
***A. abyssinica* Hochst.**												
k-15436	100.0	0.0	25.0	1.4	42.0	4.2	59.5	4.9	19.5	1.9	130.0	113.1
k-15437	127.5	10.6	26.5	0.7	50.5	6.4	59.5	13.4	18.8	3.7	137.5	74.2
k-15438	122.5	10.6	25.5	4.9	43.0	4.2	59.5	24.7	19.0	0.1	102.5	102.5
***A. byzantina* C.K.**												
k-15368	97.5	10.6	17.3	0.4	16.5	0.7	30.5	7.8	48.6*	2.8	245.0	35.4
k-15386	107.5	17.7	17.8	1.1	31.5	7.8	48.0	17.0	41.6*	0.4	267.5	60.1
k-15397	125.0	14.1	19.5	0.7	42.5	4.9	59.0	2.8	33.2	4.0	152.5	81.3
k-15435	112.5	3.5	19.3	0.4	18.5	4.9	32.5	10.6	42.6*	1.1	250.0	70.7
k-15445	112.5	24.7	19.3	2.5	25.5	2.1	45.5	3.5	47.5*	3.5	240.0	7.1
k-15450	130.0	21.2	20.0	1.4	30.0	2.8	53.5	9.2	40.3*	0.4	292.5	102.5
k-15464	125.0	0.0	19.8	1.1	35.0	2.8	57.0	2.8	36.7	3.4	365.0	190.9
k-15465	110.0	0.0	19.0	0.0	42.5	0.7	64.0	1.4	36.4	3.4	457.5*	173.2
k-15470	100.0	14.1	17.8	0.4	28.5	4.9	52.0	12.7	43.2*	1.1	342.5	81.3
k-15471	102.5	10.6	19.5	0.7	40.0	2.8	64.5	12.0	38.2*	0.8	325.0	176.8
k-15472	102.5	17.7	17.5	1.4	31.5	2.1	63.5	9.2	44.0*	4.0	192.5	236.9
k-15474	132.5	3.5	21.3	0.4	42.5	0.7	57.0	2.8	35.8	4.5	132.5	95.5
k-15475	107.5	10.6	21.5	2.1	25.0	2.8	46.0	5.7	41.9*	0.6	330.0	141.4
k-15481	105.0	7.1	17.3	0.4	22.0	4.2	35.0	9.9	47.2*	2.0	292.5	109.6

VIR Catalog No	PH, cm		LP, cm		NSP, pcs		NG, pcs		W1000, g		SP, g/m²	
	Mean	SD	Mean	SD	Mean	SD	Mean	SD	Mean	SD	Mean	SD
k-15482	100.0	14.1	16.3	1.1	18.5	0.7	36.0	1.4	46.9*	1.6	287.5	60.1
k-15484	97.5	17.7	15.0	0.0	16.5	0.7	23.5	4.9	52.1*	0.8	212.5	17.7
k-15485	97.5	17.7	14.8	1.1	16.0	1.4	30.0	1.4	46.5*	0.1	270.0	21.2
k-15486	100.0	21.2	15.5	0.7	19.0	0.0	33.0	4.2	45.9*	0.4	220.0	28.3
k-15488	90.0	14.1	14.0	0.7	16.5	0.7	26.5	0.7	48.2*	3.8	120.0	14.1
k-15489	67.5	17.7	12.8	0.4	14.5	2.1	25.0	2.8	42.5*	4.2	110.0	28.3
***A. strigosa* Schreb.**												
k-15478	157.5	10.6	20.0	0.0	56.5	4.9	55.5	3.5	21.6	2.0	95.0	63.6
k-15479	147.5	3.5	22.0	0.0	51.0	5.7	51.5	4.9	22.8	0.8	76.5	75.7
k-15480	137.5	10.6	19.3	0.4	43.5	0.7	39.0	0.0	22.5	0.6	132.5	31.8

* sources of economically valuable traits (W1000: above 38.1 g; SP: above 375.0 g/m^2).

Table 5. Statistical significance of the effect produced by the species that harbors oat accessions, individual characteristics of accessions (VIR catalogue number), year of reproduction, and their combinations on the content of main quality indicators (oil, protein, starch, and *β*-glucans) in the grain of *Avena* L. cultivars from the VIR collection.

Effect	Protein	Oil	Starch	*β*-Glucans
**Catalog (Species)**	**0.000**	**0.000**	**0.000**	**0.000**
**Year**	**0.000**	**0.000**	**0.000**	**0.000**
**Species**	**0.000**	**0.000**	**0.000**	**0.000**
**Catalog*Year*Species**	**0.000**	**0.000**	**0.000**	**0.000**
**Year*Species**	**0.000**	**0.000**	**0.000**	**0.000**

* Differences highlighted in bold ($p < 0.05$).
